# Maintaining Quality of Care among Dialysis Patients in Affected Areas after Typhoon Morakot

**DOI:** 10.3390/ijerph18147400

**Published:** 2021-07-11

**Authors:** Chia-Ming Chang, Tzu-Yuan Stessa Chao, Yi-Ting Huang, Yi-Fang Tu, Tzu-Ching Sung, Jung-Der Wang, Hsin-I Shih

**Affiliations:** 1 Department of Geriatrics & Gerontology, National Cheng Kung University Hospital, Tainan 70403, Taiwan; 10108040@gs.ncku.edu.tw; 2 School of Medicine, College of Medicine, National Cheng Kung University, Tainan 70101, Taiwan; alage@gs.ncku.edu.tw (Y.-T.H.); nckutu@gmail.com (Y.-F.T.); 3 Department of Urban Planning, National Cheng Kung University, Tainan 70101, Taiwan; tychao@mail.ncku.edu.tw; 4 Department of Pediatrics, National Cheng Kung University Hospital, College of Medicine, National Cheng Kung University, Tainan 70403, Taiwan; 5 School of Medicine for International Students, I-Shou University, Kaohsiung 82445, Taiwan; vivian1223@isu.edu.tw; 6 Department of Public Health, College of Medicine, National Cheng Kung University, Tainan 70101, Taiwan; jdwang121@gmail.com; 7 Department of Emergency Medicine, National Cheng Kung University Hospital, College of Medicine, National Cheng Kung University, Tainan 70403, Taiwan

**Keywords:** disaster, typhoon, flood, elderly, dialysis, end-stage kidney disease

## Abstract

Natural disasters have negative health impacts on patients who need dialysis in affected areas. Severely affected areas are usually rural, with limited basic infrastructure and a population without optimal dialysis-specific care after a disaster. A population-based longitudinal case–cohort study enrolled 715,244 adults from the National Health Insurance Registry who lived in areas affected by a major natural disaster, Typhoon Morakot, in 2009. The observation period was from 2008 to 2011. A total of 13,268 patients (1.85%) had a history of end-stage renal disease (ESRD). Of the ESRD patients, 1264 patients (9.5%) received regular dialysis. Only eight patients missed dialysis sessions in the first month after the disaster. Compared to the moderately affected areas, the incidences of acute cerebrovascular and cardiovascular diseases were higher in patients in severely affected areas. Male dialysis patients aged 45–75 years had a higher mortality rate than that of the general population. Among the affected adults receiving regular dialysis, patients with diabetes (adjusted hazard ratio (aHR): 1.58, 95% confidence interval (CI): 1.20–2.08) or a history of cerebrovascular disease (aHR: 1.58, 95% CI: 1.12–2.21), chronic obstructive pulmonary disease (COPD) or asthma (aHR: 1.99, 95% CI: 1.24–3.17) in moderately affected areas had significantly elevated mortality rates. Additionally, among dialysis patients living in severely affected areas, those with a history of cerebrovascular disease (aHR: 4.52 95% CI: 2.28–8.79) had an elevated mortality rate. Early evacuation plans and high-quality, accessible care for cardiovascular and cerebrovascular diseases are essential to support affected populations before and after disasters to improve dialysis patients’ health outcomes.

## 1. Introduction

Climate-associated heavy precipitation events and floods have increased in recent decades [1,2]. Increasingly variable rainfall patterns have increased the frequency and intensity of flooding, which has had substantial impacts on human health and heightened the risk of health effects in those with chronic comorbidities, communicable diseases, and stress [3,4,5]. Heavy floods and associated mudslides can cause substantial damage to basic infrastructure (electrical utilities, backup generators, and structural damage) and lead to massive economic and personal losses in affected communities [6,7,8]. Therefore, efficient large-scale coordination is required to provide urgently needed humanitarian aid and continuous care relief programs [9,10,11] to support affected areas.

Patients with chronic comorbidities need to maintain a delicate balance of care to prevent complications during and after disasters. Patients with end-stage renal disease (ESRD) on dialysis have a complex sociomedical situation and are dependent on technology and infrastructure, such as transportation, electricity, and water, to sustain their lives [8,12,13,14,15]. Compared to the unaffected areas, many affected areas are usually resource-deprived rural areas and are more likely to experience an inequitably high burden due to healthcare disparities such as the inadequate provision of basic healthcare services that arise from the relatively limited numbers of medical facilities, providers, and specialists and the accompanying lack of technical innovations and health promotion programs [16]. Affected communities, especially those that are severely affected and in rural areas, experience many disparities, each of which poses a barrier to achieving a timely response to and complete recovery from a disaster.

**Figure A1:**
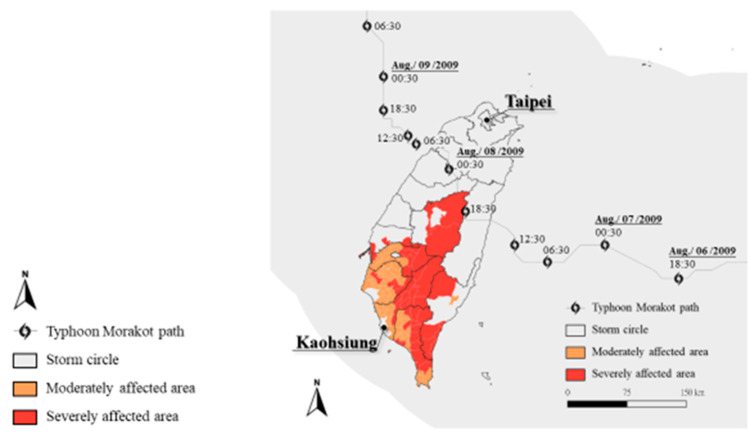
Affected areas and path of Typhoon Morakot.

A major natural disaster, Typhoon Morakot in 2009, affected the West Pacific Region and resulted in massive rainfall in Taiwan, peaking at 2777 mm (109.3 in). This extreme amount of rain triggered massive mudflows and severe flooding throughout southern Taiwan, causing 702 deaths and roughly USD 6.76 billion in financial losses [17,18]. Southern and eastern Taiwan, including six counties and cities and 168 townships, were stricken by Typhoon Morakot and listed as severely affected areas (Appendix A Figure A1). Nearly 1000 houses were reported to have collapsed. All connecting roads, bridges, and railways of the severely affected areas were destroyed by mudslides and floods and rebuilt six months after Typhoon Morakot. One million households lost power and tap water supply for at least one week. All primary healthcare centers in the severely affected areas discontinued medical services for at least two weeks. Emergency medical service teams were deployed to temporary shelters in severely affected areas to provide primary medical services [19,20]. The population living in neighborhoods affected by severe floods and mudslides needed to relocate to temporary shelters for approximately six months to one year. Residents who lived in the affected areas were identified by the Ministry of Health and Welfare in Taiwan and received partial reimbursement for medical expenses for three months. Primary medical services were established in the temporary shelters in the severely affected areas for at least six months. The affected population requiring hemodialysis was relocated to unaffected areas to enable them to undergo regular hemodialysis. We conducted a longitudinal population-based follow-up cohort study monitoring the health parameters of adult dialysis patients to compare and evaluate health outcomes in areas affected at different levels of severity following Typhoon Morakot.

## 2. Materials and Methods

### 2.1. Data Source

Data from the Taiwan National Health Insurance (NHI) Database from the National Health Research Institute (NHRI) were analyzed. The NHRI data included residents’ demographic data, medications, treatments (including operations), and disease diagnoses (based on the International Classification of Diseases, Ninth Revision, Clinical Modification [ICD-9-CM]) in different levels of healthcare facilities. The “National Health Insurance Reimbursement for Medical Expenses after Typhoon Morakot” regulation was implemented after Typhoon Morakot. The NHI Bureau identified residents who had lived in the affected areas before the disaster and provided partial reimbursement of their medical expenses for 3 months.

### 2.2. Study Design

A population-based longitudinal case–cohort study was conducted. In total, historical data from 715,244 adults who had lived in the affected areas were obtained for this study. Adult patients with healthcare facility visit records were analyzed. The index date was 7 August 2009, when Typhoon Morakot made landfall in Taiwan.

**Figure 1:**
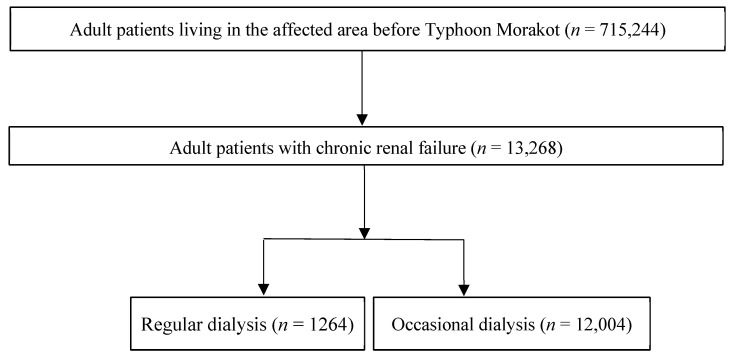
Flow diagram of patient enrollment.

Patients with underlying medical comorbidities, including hypertension, diabetes mellitus (DM), asthma, chronic heart failure, chronic obstructive pulmonary disease (COPD), liver cirrhosis, neoplasms and chronic kidney disease (CKD), that were recorded before the index date, were selected (selected ICD-CM 9: Appendix A Table A1). Patients aged more than 18 years old with illnesses related to dialysis (hemodialysis [HD] or peritoneal dialysis [PD]) therapy who had received dialysis for more than three consecutive months before the index date were enrolled in the dialysis cohort (Figure 1). The observation period of the study was from January 2008 to December 2011.

All medical service utilization records after the index date were reviewed. The calculated Charlson Comorbidity Index (CCI) scores indicated the severity of these patients’ comorbidities. Patients’ socioeconomic statuses were based on their income records, as reported to the NHI Bureau.

Hospitalization records after Typhoon Morakot were also analyzed to evaluate health comorbidities, including acute pulmonary edema, acute ischemic heart diseases (IHD), acute cerebrovascular diseases (CVD), heart failure, peripheral arterial occlusive diseases (PAOD), common infections with known pathogens, central nervous system (CNS) infections, lower respiratory tract infections, urinary tract infections, skin and soft tissue infections, and trauma and injury (selected ICD-CM 9: Appendix A Table A1).

The validated definition of mortality was adapted from the NHI database and was based on the insurance status of affected adults enrolled in our study. Mortality was defined in enrolled adults who were withdrawn from insurance due to death or who received a critical discharge with a diagnosis of one of the twenty most common causes of death (ICD-9-CM) according to the National Statistics in Taiwan and lacked medical records after the withdrawal date, were missing for more than six months, or were disqualified as insurance applicants in the NHI program for reasons such as immigration or the expiration of the duration of stay of noncitizens [21,22,23]. All causes of death for the affected adults enrolled in this study between August 2009 and December 2011 were investigated in the survival and hazard ratio analysis. The mortality of dialysis patients in the general population was obtained from the Annual Report on Kidney Disease in Taiwan of the Taiwan Society of Nephrology [24].

### 2.3. Social and Spatial Analysis

The township classifications and metropolitan areas in Taiwan were adapted from the 2005 Taiwan Social Change Survey, which tracks and provides long-term societal trends and developments in each town or metropolitan area in six prospective areas through nationally representative survey data. Three different types of towns or metropolitan areas (urban, suburban, and rural) were included [25].

### 2.4. Statistical Analysis

The distributions of the study population were analyzed according to demographics and disease history data and mortality. To evaluate differences among the study groups, the chi-squared test was used for categorical variables, and Student’s *t* test was used for continuous variables. Age and sex were adjusted for further model analysis. Hazard ratios for all-cause mortality were estimated with Cox proportional hazards models with backward elimination for survival time (time-to-event) to compare the relative difference between populations located in severely and moderately affected areas. All statistical tests were 2-sided, and *p*-values less than 0.05 were considered statistically significant. All data management and statistical analysis was performed with SAS 9.4 software (SAS Institute, Cary, NC, USA).

### 2.5. Ethics Issues

This study was performed in accordance with the principles of the Declaration of Helsinki and the Declaration of Taipei [26]. Patients’ personal information was encrypted to protect their privacy. The electronic databases were decoded for research. Informed consent was waived and approved by the institutional review board (IRB) of the study hospital (IRB No: A-ER-103-176).

## 3. Results

In total, 715,244 affected adult files were identified, among whom 13,268 patients (1.85%) had a history of ESRD. Of the ESRD patients, 1264 patients (9.5%) received regular dialysis. Females were slightly more represented among the enrolled dialysis patients (689/1264, 55%). The demographic characteristics of the study population in areas affected to different degrees are presented in Table 1. The majority of the dialysis patients received hemodialysis (severely affected area: 80% vs. moderately affected area: 88%). Patients on regular dialysis in the severely affected area were older (62.69 (13.27) vs. 62.37 (12.45) years), consisted of a higher proportion of individuals living in rural areas (61% vs. 23%), and included a higher proportion of individuals with multiple underlying diseases (CCI > 2: 77% vs. 71%) than those in the moderately affected area. In addition, the group receiving regular dialysis in the severely affected area included higher proportions of individuals with underlying chronic diseases, such as DM (42% vs. 33%) and hypertension (44% vs. 29%).

Missed dialysis sessions in the first month after Typhoon Morakot were analyzed. Of all the adults receiving regular dialysis before Typhoon Morakot and living in the affected areas, eight (8/1105, 0.73%) missed HD sessions, and none missed PD sessions in August 2009. Three individuals living in the severely affected areas (3/8, 37.5%) missed HD sessions. Six of the eight patients who missed dialysis living in the moderately affected areas died within 30 days after Typhoon Morakot.

The analysis of hospitalization after matching in differently affected ESRD adults receiving regular dialysis before and after Typhoon Morakot is summarized in Table 2. After matching for age and sex, the incidences of acute pulmonary edema (moderately affected areas: 43 vs. 15; severely affected areas: 55 vs. 18 per 1000 population, per year), acute IHD (moderately affected areas: 134 vs. 113; severely affected areas: 180 vs. 111 per 1000 population, per year), acute CVD (moderately affected areas: 127 vs. 111; severely affected areas: 160 vs. 127 per 1000 population, per year), and PAOD (moderately affected areas: 6 vs. 5; severely affected areas: 11 vs. 0 per 1000 population, per year) were lower among the affected adults on regular dialysis after than before Typhoon Morakot; however, the incidence of infection (moderately affected areas: 436 vs. 441; severely affected areas: 413 vs. 425 per 1000 population, per year) in the affected adults on regular dialysis was higher in both the moderately and severely affected areas after Typhoon Morakot. Furthermore, the incidence of infections (413 vs. 425 per 1000 population per year) and injury (138 vs. 142 per 1000 population per year) in the affected adults on regular dialysis was relatively higher in the severely affected areas.

**Figure 2:**
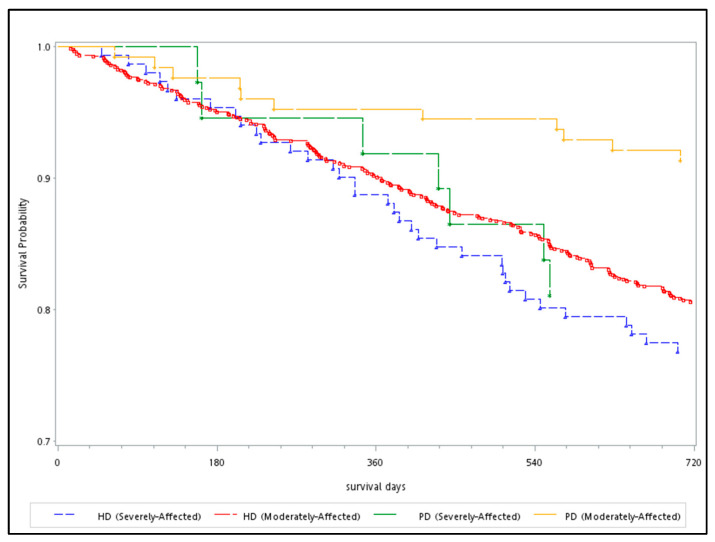
Survival analysis of adults with ESRD living in areas affected to different degrees by Typhoon Morakot.

Figure 2 shows long-term survival stratified by dialysis type among adults living in the affected areas before and after Typhoon Morakot, i.e., from 2008 to 2010. Compared to patients receiving HD, patients receiving PD had a higher survival rate within one to two years. Regardless of whether patients received HD or PD, patients living in severely affected areas had a lower survival rate than those living in moderately affected areas (log-rank test, *p* = 0.003).

The mortality rates of the dialysis patients living in areas affected to different degrees were compared (Table 3). Compared to the dialysis patients nationwide, the mortality rate of dialysis patients in moderately affected areas was lower among both females (moderately affected areas: 75.9 [first year] vs. national levels: 105 [2009] and 112 [2010] deaths per thousand regular dialysis patients; 82.1 [1–2 years] vs. national levels: 112 [2010] and 115 [2011] deaths per thousand regular dialysis patients) and males (moderately affected areas: 113 [first year] vs. national levels: 117 [2009] and 122 [2010] deaths per thousand regular dialysis patients; 116 [1–2 years] vs. national levels: 122 [2010] and 126 [2011] deaths per thousand regular dialysis patients). However, a statistical interaction existed between gender and area. Compared to the dialysis patients nationwide, the mortality rate among dialysis patients in the severely affected area was lower among females (severely affected areas: 92 [first year] vs. national levels: 105 [2009] and 112 [2010] deaths per thousand regular dialysis patients; 101 [1–2 years] vs. national levels: 112 [2010] and 115 [2011] deaths per thousand regular dialysis patients) and higher among males (severely affected areas: 127 [first year] vs. national level: 117 [2009] and 122 [2010] deaths per thousand regular dialysis patients; 174 [1–2 year] vs. national level: 122 [2010] and 126 [2011] deaths per thousand regular dialysis patients). We performed further stratification by age. Unlike female elderly dialysis patients, male elderly dialysis patients living in the moderately affected and severely affected areas had a relatively higher mortality rate than the national annual mortality rate (moderately affected areas: 169 and 246 [first year] vs. national levels: 139 and 247 [2009] and 141 and 258 [2010] deaths per thousand regular dialysis patients; 144 and 230 [1–2 year] vs. national levels: 141 and 258 [2010] and 141 and 277 [2011] deaths per thousand regular dialysis patients; severely affected areas: 130 and 91 [first year] vs. national levels: 139 and 247 [2009] and 141 and 258 [2010] deaths per thousand regular dialysis patients; 300 and 300 [1–2 year] vs. national levels: 141 and 258 [2010] and 141 and 277 [2011] deaths per thousand regular dialysis patients).

To determine the important risk factors and eliminate confounding factors for mortality among dialysis adults in severely and moderately affected areas, a multivariate Cox proportional hazards regression model with backward selection, adjusted by sex and age, was constructed (Table 4). Among dialysis adults in the moderately affected areas, patients with DM (adjusted HR: 1.58, 95% CI: 1.20–2.08, *p* < 0.0001), a history of cerebrovascular disease (adjusted HR: 1.58, 95% CI: 1.12–2.21, *p* < 0.0001), and COPD and asthma (adjusted HR: 1.99, 95% CI: 1.24–3.17, *p* < 0.0001) had a significantly elevated risk of mortality. Additionally, among dialysis adults living in severely affected areas, patients with a history of cerebrovascular disease (adjusted HR: 4.52 95% CI: 2.28–8.79, *p* < 0.0001) had a higher risk of mortality.

## 4. Discussion

The results of this study suggested that adults on regular dialysis in affected areas were at increased risk for hospitalization due to infection and injury, but not due to acute pulmonary edema, acute ischemic heart diseases, acute cerebrovascular diseases, or PAOD after Typhoon Morakot. The survival analysis suggested that the all-cause mortality rate was higher among adult patients on dialysis living in severely affected areas than among those in moderately affected areas. Concerning dialysis patterns and affected areas, of all the adults on regular dialysis living in the affected areas, those receiving PD in the moderately affected areas had the lowest mortality rate, followed by those receiving PD in the severely affected areas, those receiving HD in the moderately affected areas and those receiving HD in the severely affected areas. Relatively higher mortality rates were observed in male dialysis patients living in both moderately and severely affected areas.

Caring for dialysis patients during and after disasters is challenging. Lack of access to clean water and power, closures of dialysis centers and primary healthcare facilities, and difficulties with transportation limit patient access to dialysis after disasters [14,27]. Missed dialysis sessions and poor control of underlying diseases were the most common causes of ED visits and hospitalizations. After Hurricane Katrina, only 72% of patients did not miss their dialysis [28]. Our study suggested that limited patients (<1%) receiving HD, and no patients receiving PD missed dialysis sessions. The emergency response plan based on the Disaster Prevention and Protection Act in 2009 defined vulnerable groups, such as the elderly population, dialysis patients, pregnant women, and disabled people requiring special care, who need early evacuations before and during disasters. Dialysis patients are given priority for early evacuation in mudslides and typhoons in Taiwan before disasters [29]. Although catastrophic floods, mudslides, and the destruction of roads resulted from Typhoon Morakot, the majority of dialysis patients living in the affected area were evacuated early, ensuring that they did not miss a dialysis session after the disaster. In addition, dialysis patients’ information in each dialysis center has been uploaded to the cloud database to evaluate the quality of dialysis patients since 2003. After Typhoon Morakot, basic services such as power, clean water, and medical resource supplies for primary healthcare facilities were the priority. Most primary medical care services returned to the previous levels within one month [19].

The Sendai framework for disaster risk reduction 2015–2030 aims to achieve substantial reductions in disaster risks and losses by enhancing the resilience of national health systems. Corresponding efforts include strengthening the development and implementation of inclusive policies and social safety-net mechanisms and ensuring access to basic healthcare services, with the ultimate aim of eradicating poverty [30]. The World Health Organization (WHO) Health Emergency and Disaster Risk Management (EDRM) framework emphasizes that health systems should work on risk-based approaches, comprehensive emergency management (across prevention, preparedness, readiness, response and recovery), all-hazards approaches, people- and community-centered approaches, multisectoral and multidisciplinary collaborations, whole-health system-based approaches, and ethical considerations. To be well prepared for foreseeable catastrophic natural events caused by climate change, laws and policies have been enacted, including the most important “Spatial Planning Act 2016” [31] (state of the new climate-adaptive and environmentally oriented land use policy) in Taiwan. In 2018, the Ministry of the Interior announced the implementation of the National Spatial Plan in accordance with the law and further emphasized prioritizing the conservation of environmentally sensitive areas as well as important public facilities, including medical services in rural and coastal areas. In addition, the United Nations Sustainable Development Goals (SDGs) [32] (United Nations, 2015) form the basis of spatial plans at all levels to ensure that health inequality and urban–rural gaps are taken into account in future land-use policies. The Ministry of Health and Welfare also follows the policies and strategies of the Central Disaster Prevention and Response Council and has further established emergency response surveillance systems and guidelines for healthcare facilities to relieve the impacts of disasters [29,33,34].

Collecting sufficient information to maintain optimal care of dialysis patients after major disasters is complicated. Studies after Hurricane Katrina suggested the importance of a comprehensive database that includes information about comorbidities and hemodialysis parameters [35]. Many studies have used the Medicaid and Medicare databases to assess dialysis patients after hurricanes [27]. The Medicaid and Medicare system does not cover patients who are not elderly or those with relatively higher socioeconomic status. Our study used the NHI database to obtain data pertaining to dialysis patients living in affected areas. Comprehensive databases associated with universal healthcare systems that contain healthcare information from before and after the disaster are beneficial for long-term health outcome follow-up studies. Comprehensive universal healthcare systems are more readily able to track patients’ locations, illness diagnoses, medications, treatments, and prognoses. This two-year follow-up study found that dialysis patients received adequate care for comorbid cardiovascular diseases (ischemic heart diseases, cerebrovascular diseases and PAOD). The primary care system maintained the necessary quality of care in the affected population during and after the disaster. The current recommendations for dialysis patients during disasters include early evacuation, arrangements for ongoing dialysis sessions, and provision of information to the database [36]. High-quality and accessible universal healthcare systems maintain care quality and support affected populations after disasters and are regarded as an essential aspect of community resilience [37]. Previous studies have suggested that Taiwan’s NHI has greatly increased the utilization of outpatient and inpatient services [38,39]. This increased utilization of health services reduces mortality and leads to a better general health status of the Taiwanese elderly population, even in rural areas [38,39,40]. The NHI-based healthcare system in Taiwan is considered to have good accessibility, comprehensive coverage, short waiting times, low cost, and a high coverage rate [41]. This healthcare system provides convenient and affordable care for socially and economically disadvantaged patients. Similar to a previous study [42], the majority of the dialysis patients living in the affected areas in our study were socially deprived. ESRD is listed as one of the catastrophic illnesses in Taiwan’s NHI system; therefore, patients with ESRD receive copayment exemptions. Dialysis patients in the affected areas also received further partial reimbursement of their medical expenses for 3 months. Medical expense reimbursement should ensure that dialysis patients in affected areas are able to maintain basic care qualities and prevent serious, costly consequences. Universal healthcare systems might substantially reduce socioeconomic inequalities in access to and the quality of primary care but are associated with only modest reductions in disparities in healthcare outcomes [43,44,45].

Social demographics such as socioeconomic status, sex, geographic location, income, and education are still important factors affecting the accessibility of health services, especially in rural areas [46]. Previous studies in Canada have suggested that rural residents have reduced access to basic stroke care, such as computed tomography, thrombolysis, a stroke unit, a stroke prevention clinic, or inpatient rehabilitation once they have had a stroke, and therefore experience relatively more severe outcomes, including disability [47,48]. Our study also found that dialysis patients living in severely affected areas had a higher risk of mortality than patients in moderately affected areas. Specialty and sub-specialty healthcare services are usually less likely to be available in rural areas, and rural areas are less likely to offer specialized and highly sophisticated or high-intensity care than suburban or urban areas. Communities in the severely affected areas were more likely to be characterized by low socioeconomic levels. Stroke patients receiving dialysis need more careful treatment than other dialysis patients and have substantially increased hospitalization and mortality rates, highlighting the importance of access to medical care. Although relocation villages were established with permanent houses and healthcare centers within one year after Typhoon Morakot in the severely affected areas, the accessibility of public transportation to advanced cardiovascular and cerebrovascular and rehabilitation care centers was limited. Such limitations on accessibility had negative impacts on adults with cardiovascular diseases, resulting in negative health outcomes.

Some limitations also need to be mentioned. Firstly, the data were collected from a claim database, and the database did not include unaffected adults as a reference group to obtain associated risk factor information. These are important potential confounding factors of chronic diseases in affected populations [22,23]. Sex and age were adjusted for in the two groups to minimize the effects of comorbidities in the elderly population. Secondly, the claim data did not include some lifestyle information, such as smoking habits, alcohol consumption, body mass index values, physical activity, and family histories. Although the underlying diseases between the two groups were different, the CCI scores were similar between the two groups. Thirdly, the database did not include an unaffected population as a reference group that could be used to evaluate baseline health status before Typhoon Morakot. However, we compared mortality with that in the national dialysis patient database, which was considered the reference group, to precisely evaluate the effects of Typhoon Morakot at different levels in the affected population. Additionally, we chose the primary medical facilities most commonly used by patients in affected areas before the disaster as their living pace. It was difficult to locate all the affected individuals’ living places precisely because some patients visiting these medical facilities might not have been residing in the same area that they did before the disaster. Furthermore, the levels of township development and the proportions of elderly individuals in the populations were based on the 2005 Taiwan Social Change Survey (Round 5), which primarily considered socioeconomic change and township development in Taiwan. This is the first study to apply this survey data to evaluate relationships between socioeconomic factors and ESRD. However, data from the national census, such as the proportion of elderly individuals in the population and household income, were acquired to achieve optimal validity.

## 5. Conclusions

In conclusion, our study shows that Typhoon Morakot did not have significant negative effects on the health status of dialysis patients in either the short term or long term after Typhoon Morakot. Few dialysis patients missed their dialysis sessions after the disaster. More hospitalizations occurred in the affected population living in the severely affected areas. Compared to the national data, the mortality rate was higher among the male population in severely affected areas. Dialysis patients receiving PD had a lower risk of mortality. Dialysis patients with other comorbidities, such as diabetes, a history of CVD, and COPD and asthma, had a higher hazard of mortality. High-quality, accessible universal healthcare systems are essential for supporting dialysis populations affected by disasters, especially among those living in severely affected areas. Further long-term spatial and socioeconomic analyses of the health of affected dialysis populations might be conducted to support the development of more resilient communities.

## Figures and Tables

**Table 1:** Demographic and clinical characteristics of adults with ESRD living in areas affected by Typhoon Morakot to differing degrees of severity.

Characteristics	Adult Patients with ESRD (2008/1–2009/7) *n* = 13,268				
	Regular Dialysis (*n* = 1264)				
	Moderately Affected Areas		Severely Affected Areas		
	(*n* = 1076)		(*n* = 188)		Chi-Squared Test
	No.	%	No.	%	*p*-Value
Sex					
Male	496	46.10	79	42.02	0.30
Age (years)					
Mean (SD) *	62.37 (12.45)		62.69, 13.27		
Age group					
Elderly	481	44.70	94	50.00	0.18
Socioeconomic status (USD/month)					
Poor < 750 USD	647	60.13	139	73.94	0.0014
Location					
Urban	245	22.77	0	0.00	<0.0001
Suburban	586	54.46	73	38.83	
Rural	245	22.77	115	61.17	
CCI ^†^					
0	-	-	-	-	
1–2	309	28.72	44	23.40	0.13
>2	767	71.28	144	76.60	
Underlying diseases					
DM	352	32.71	79	42.02	0.01
Hypertension	309	28.72	82	43.62	<0.0001
Heart disease	260	24.16	36	19.15	0.13
COPD and asthma	47	4.37	9	4.79	0.80
Liver cirrhosis	32	2.97	6	3.19	0.87
Neoplasms	111	10.32	15	7.98	0.32
Dialysis					
Hemodialysis	949	88.20	151	80.32	0.003
Peritoneal dialysis	127	11.80	37	19.68	

* Standard deviation; ^†^ Charlson Comorbidity Index; DM: diabetes mellitus; COPD: chronic obstructive pulmonary disease.

**Table 2:** Hospitalizations of dialysis patients living in the affected areas before and after Typhoon Morakot.

Characteristics	Regular Dialysis Patients before the Disaster (*n* = 1264)											
	Moderately Affected Areas (*n* = 1076)						Severely Affected Areas (*n* = 88)					
	Before Aug/06/2008–Aug/07/2009			After Aug/08/2009–Aug/07/2010			Before Aug/06/2008–Aug/07/2009			After Aug/08/2009–Aug/07/2010		
	No.	Person-Year	Incidence ^†^	No.	Person-Year	Incidence ^†^	No.	Person-Year	Incidence ^†^	No.	Person-Year	Incidence ^†^
Acute pulmonary edema	44	1035	42.51	14	946	14.80	10	183	54.64	3	165	18.17
Acute ischemic heart diseases	116	867	133.79	81	719	112.61	27	150	180.00	13	117	111.12
Acute cerebrovascular diseases	113	888	127.25	83	745	111.44	26	163	159.51	17	134	127.04
Infection	274	628	436.31	150	340	440.78	43	104	413.46	25	59	424.64
PAOD	6	1070	5.61	5	1015	4.92	2	188	10.64	0	178	0.00
Injury (800–859)	169	907	186.33	118	695	169.72	22	160	137.50	19	134	141.53

Before: New cases between August 06/2008 and August 07/2009; After: New cases between August 08/2009 and August 07/2010; ^†^: person-time incidence rate (per 1000 population, per year); PAOD: peripheral arterial occlusive diseases.

**Table 3:** Mortality in dialysis patients living in different places before and after Typhoon Morakot.

Survival, Years	National Dialysis Data	Moderately Affected Areas (*n* = 1076)						Severely Affected Areas (*n* = 188)						National Dialysis Data		
	Yearly	<1 Year (2009/08–2010/07)			1–2 Year (2010/08–2011/07)			<1 Year (2009/08–2010/07)			1–2 Year (2010/08–2011/07)			Yearly		
	Mortality ^†^	(*n* = 100)			(*n* = 95)			(*n* = 20)			Mortality ^†^			Mortality ^†^		
	2008	No	Cases	Mortality ^†^	No	Cases	Mortality ^†^	No	Cases	Mortality ^†^	No	Cases	Mortality ^†^	2009	2010	2011
Sex																
Female	109	44	580	75.9	44	536	82.1	10	109	91.7	10	99	101.0	105	112	115
Male	126	56	496	112.9	51	440	115.9	10	79	126.6	12	69	173.9	117	122	126
Age group																
20–44	32	1	92	10.9	0	91	-	3	20	150.0	0	17	-	26	29	31
45–64	67	27	503	53.7	36	476	75.6	6	74	81.1	6	68	88.2	62	66	66
65–74	133	40	312	128.2	27	272	99.3	7	60	116.7	9	53	169.8	123	126	128
75+	251	32	169	189.3	32	137	233.6	4	34	117.6	7	30	233.3	238	244	254
Females, age group																
20–44	26	0	43	-	0	43	-	1	10	100.0	0	9	-	22	28	31
45–64	54	16	279	57.3	16	263	60.8	2	39	51.3	3	37	81.1	49	55	55
65–74	121	16	170	94.1	10	154	64.9	4	37	108.1	3	33	90.9	110	114	116
75+	238	12	88	136.4	18	76	236.8	3	23	130.4	4	20	200.0	231	233	236
Males, age group																
20–44	38	1	49	20.4	0	48	-	2	10	200.0	0	8	-	30	29	31
45–64	81	11	224	49.1	20	213	93.9	4	35	114.3	3	31	96.8	74	76	75
65–74	148	24	142	169.0	17	118	144.1	3	23	130.4	6	20	300.0	139	141	141
75+	267	20	81	246.9	14	61	229.5	1	11	90.9	3	10	300.0	247	258	277

^†^ Mortality: death per thousand regular dialysis patients.

**Table 4:** Hazard ratio for all-cause mortality in adults on dialysis in moderately and severe affected areas after Typhoon Morakot.

	Moderately Affected Areas ^†^ (*n* = 1076)				Severely Affected Areas ^†^ (*n* = 188)			
	Univariate Analysis		Multivariate Analysis Backward Elimination		Univariate Analysis		Multivariate Analysis Backward Elimination	
	HR	95% CI	aHR	95% CI	HR	95% CI	aHR	95% CI
Location (Ref = Nonrural)								
Rural	1.15	0.84–1.56			0.85	0.48–1.51		
Diabetes (Ref = No)								
Yes	1.58 *	1.21–2.07	1.58 *	1.20–2.08	1.05	0.59–1.86		
Hypertension (Ref = No)								
Yes	1.08	0.81–1.45			0.92	0.52–1.63		
Arrhythmia history (Ref = No)								
Yes	1.43	0.78–2.63			0.79	0.11–5.76		
Cerebrovascular disease events (Ref = No)								
Yes	2.06 *	1.48–2.88	1.58 *	1.12–2.21	4.45 *	2.26–8.76	4.52 *	2.29–8.92
Ischemic heart disease events (Ref = No)								
Yes	1.51 *	1.13–2.01			1.32	0.67–2.59		
PAOD (Ref = No)								
Yes	1.55	0.77–3.14			—	—		
Two cardiovascular conditions (Ref = No)								
Yes	1.51 *	1.12–2.02			1.60	0.86–2.98		
>2 cardiovascular conditions (Ref = No)								
Yes	1.75 *	1.17–2.62			2.42	0.87–6.74		
COPD and asthma (Ref = No)								
Yes	2.57 *	1.62–4.07	1.99 *	1.25–3.18	2.24	0.80–6.24		
Liver cirrhosis (Ref = No)								
Yes	1.74	0.92–3.28			1.50	0.36–6.18		
Neoplasms (Ref = No)								
Yes	1.02	0.66–1.57			1.00	0.36–2.78		
Injury 800–859 (Ref = No)								
Yes	0.79	0.48–1.27			1.29	0.47–3.60		
Infection (Ref = No)								
Yes	1.22	0.86–1.74			1.35	0.60–3.00		

^†^ Enrolled variables in the Cox model for cerebrovascular disease history and cerebrovascular disease history: location, DM, hypertension, CKD, COPD and asthma, liver cirrhosis, neoplasm. DM: diabetes mellitus; CKD: chronic kidney disease, COPD: chronic obstructive pulmonary disease. PAOD: peripheral arterial occlusive diseases * *p* < 0.05.

## Data Availability

The research data were provided by the NHRI (NHIRD-104-419, NHIRD-104-194 and NHIRD-103-197). This study was partially based on data from the National Health Insurance Research Database provided by the National Health Insurance Administration, Ministry of Health and Welfare and managed by the NHRI. The interpretation and conclusions contained herein do not represent those of the National Health Insurance Administration, Ministry of Health and Welfare or NHRI.

## Appendix A

**Table A1:** Selected ICD-CM9 Used for Analysis.

Diagnosis	ICD-9-CM
Hypertension	401–405
Diabetes mellitus (DM)	250
Asthma	493
Chronic heart failure	428, 410–414
Chronic obstructive pulmonary diseases (COPD)	491–496, excluding 493, 495
Liver cirrhosis	571.5
Neoplasm	140–239
Chronic kidney diseases (CKD)	585
Acute pulmonary edema	518.4
Acute ischemic heart diseases (IHD)	410–414
Acute cerebrovascular diseases (CVD)	428, 430–437
Heart failure	428
Peripheral arterial occlusive diseases (PAOD)	440.0, 440.2, 440.3, 440.8, 440.9, 443, 444.0, 444.2, 444.8, 447.8, and 447.9
Infection with known pathogens	001–139
Central nervous system (CNS) infection	320–326
Lower respiratory tract infection	480–488
Urinary tract infection	590, 595, 597, 599
Skin and soft tissue infection	680–686
Trauma and injury	850–959
